# Fast Outlier Detection Using a Grid-Based Algorithm

**DOI:** 10.1371/journal.pone.0165972

**Published:** 2016-11-10

**Authors:** Jihwan Lee, Nam-Wook Cho

**Affiliations:** 1 Department of Industrial and Management Engineering, Hankuk University of Foreign Studies, Gyunggi-do, Republic of Korea; 2 Department of Industrial and Information Systems Engineering, Seoul National University of Science and Technology, Seoul, Republic of Korea; Universita degli Studi di Catania, ITALY

## Abstract

As one of data mining techniques, outlier detection aims to discover outlying observations that deviate substantially from the reminder of the data. Recently, the Local Outlier Factor (LOF) algorithm has been successfully applied to outlier detection. However, due to the computational complexity of the LOF algorithm, its application to large data with high dimension has been limited. The aim of this paper is to propose grid-based algorithm that reduces the computation time required by the LOF algorithm to determine the k-nearest neighbors. The algorithm divides the data spaces in to a smaller number of regions, called as a “grid”, and calculates the LOF value of each grid. To examine the effectiveness of the proposed method, several experiments incorporating different parameters were conducted. The proposed method demonstrated a significant computation time reduction with predictable and acceptable trade-off errors. Then, the proposed methodology was successfully applied to real database transaction logs of Korea Atomic Energy Research Institute. As a result, we show that for a very large dataset, the grid-LOF can be considered as an acceptable approximation for the original LOF. Moreover, it can also be effectively used for real-time outlier detection.

## Introduction

As one of data mining techniques, outlier detection aims to discover outlying observations that deviate substantially from the reminder of the data. Identifying an outlier is an important task in many applications because an outlier frequently contains useful information on abnormal behavior in a system, possibly generated by a different mechanism [1–2].

Recently, the Local Outlier Factor (LOF) algorithm has been successfully applied to outlier detection [3]. The LOF algorithm is a density-based algorithm that detects the local outliers of a dataset by assigning a degree of outlierness, called the local outlier factor (LOF), to each object [4–5]. In the LOF algorithm, data points with a lower density than their surrounding points are identified as outliers [3].

As the LOF algorithm can detect “local” outliers regardless of the data distribution of normal behavior [3], it has been applied to various applications including network intrusion detection and process monitoring [6–7]. Due to the computational complexity of the LOF algorithm, however, its application to large data with high dimension has been limited. This issue can be more critical for real-time application systems.

The complexity issue can be addressed from two perspectives [8]. The computation time of LOF grows exponentially with the number of dataset dimensions *n*, called the “Curse of Dimensionality”. For high-dimensional data, the complexity frequently becomes *O*(*n*^2^)[4]. Thus, efforts have been made to reduce computational complexity related to high-dimensional data. Singular Value Decomposition (SVD) [9–10], Karhunen-Loève (KL) [11], and FastMap [12] have been proposed. Aggarwal and Yu [1] proposed a Genetic Algorithm that can determine the optimal projection for dimensional reduction in outlier detection of high-dimensional data.

The second approach relates to the computation of the k-nearest neighbors. The problem of determining the k-nearest neighbors of a data point can be formulated as follows (Kim et al., 2011): Suppose a dataset, *S* ⊂ *R*^*d*^, is composed of *n* data points in *d*-dimensional real space. For a given query object, *q* ∈ *R*^*d*^, the problem is to determine the *k* number of objects whose Euclidean distances from q are closest to the query object. The original LOF algorithm by Breunig et al. (2000) [4] computed distances from the entire dataset to the query object and sorted the distance data, resulting in large calculation time. To reduce the computation required for the k-nearest neighbors, Kim et al. [8] utilized kd-tree indexing with approximated k-nearest neighbors.

The objective of this paper is to develop a methodology that reduces the computation time required by the LOF algorithm to determine the k-nearest neighbors. An algorithm that divides the data spaces in to a smaller number of regions, called as a “grid”, is proposed. To examine the effectiveness of the proposed method, several experiments incorporating different parameters were conducted. In addition to the datasets obtained from the UCI machine-learning repository [13], a real dataset composed of database transaction logs of KAERI (Korea Atomic Energy Research Institute) was used in the experiments. The proposed method demonstrated a significant computation time reduction with predictable and acceptable trade-off errors.

## Methods

In this paper, a grid-based LOF algorithm is proposed. The proposed algorithm divides the data space into a smaller number of regions, called a grid, and calculates the LOF value of each grid. Then, the LOF value of a grid is used to determine the LOF values of the data points that belong to the grid.

The overall procedure of the proposed methodology is depicted as follows. Consider a dataset, *S* ∈ *R*^*d*^ is composed of *n* data points in d-dimensional real space. Suppose that the number of grids per dimension is set to *k*. The algorithm for computing the grid-LOF values of the data points is as follows:

1. Divide the data space of each dimension in *S* into *k* equidistant intervals. Generate total *k*^*d*^ grids over the dataset.
2. Associate each data point *x*_*i*_ ∈ *S* with one of the grid indexes, *j* = {1,…,*k*^*d*^}. If none of the data points belong to a grid, the grid is not considered.
3. For each grid *j* in the dataset, calculate the grid centroid *C*_*j*_.
4. For each grid centroid *C*_*j*_, calculate the LOF, *LOF*(*C*_*j*_). For more detailed information for computing the LOF value, please refer to [4] Breuning *et al*. (2000).
5. Determine the grid-LOFs for each data point. If *x*_*i*_ belongs to grid *j*, then *LOF*_*G*_(*x*_*i*_) = *LOF*(*C*_*j*_).

**Fig 1:**
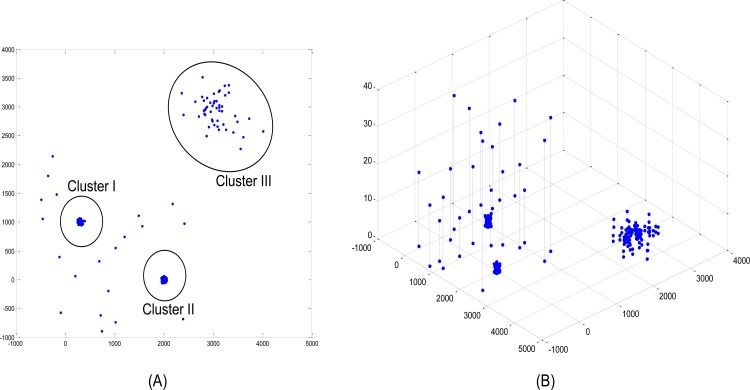
Artificial Dataset. (A) 530 data points in two dimensional space. The majority of the dataset belongs to one of the three clusters. Cluster I: Bivariate Normal distribution with mean (300,1000) with covariance 500. Cluster II: Bivariate Normal distribution with mean (2000,0) with covariance 50. Cluster III: Bivariate Normal with mean (3000,3000) with covariance 100000. The outlier point is generated by the uniform distribution. (B) LOF value of data points (MinPts = 10).

To illustrate the difference between the original LOF algorithm and the proposed algorithm, consider a two-dimensional dataset consisting of 530 data points. The overview of the data is illustrated in Fig 1A. As can be observed, the normal data points that are within ellipse boundaries were generated from three bivariate normal distributions with different means and variances. Conversely, the outliers that are outside of the ellipse boundaries were generated from a uniform distribution. Fig 1B indicates the LOFs of each data point in Fig 1A. As can be seen, the LOF values of the normal data are less than one, whereas the LOF values of the outliers are greater than five.

**Fig 2:**
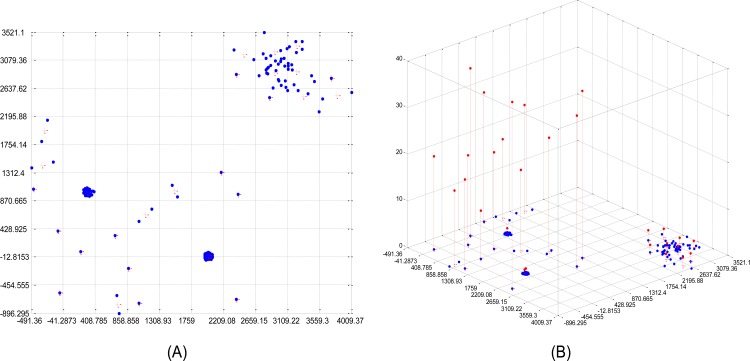
Grid-LOF values of the dataset in Fig 1. (A) Grid centroids (k = 10). (B) LOF values of Grid centroids.

Fig 2A and 2B illustrate the result of the grid-LOF method with the same data as Fig 1. As indicated in this figure, each dimension is first divided into ten equidistant intervals, generating 10^2^ grids for the data space. In contrast to the brute-force algorithm, the grid-LOF algorithm only considers the grid centroid represented by the cross mark (+) in Fig 2A. Note that the grids without data points are not considered for the LOF calculation. Fig 2B displays the LOFs of each data point obtained by the grid-LOF method. In the next section, the performance of the grid-LOF algorithm is evaluated through several real datasets.

## Experiment

Four datasets were used to compare the performance of the original LOF algorithm and the grid-LOF algorithm. All of these datasets were obtained from the UCI machine-learning repository [13]. Table 1 provides an overview of the datasets. The purpose of the experiment was to compare the performance between the original LOF algorithm and the grid-LOF algorithm. Although the grid-LOF algorithm could be more efficient than the brute-force algorithm in terms of computation time, the LOF values obtained by the grid-LOF algorithm could also lead to both Type 1 and 2 errors. However, if the quality deterioration of the LOFs with the grid method remains at an acceptable level, the use of the grid-LOF algorithm can be justified. In the experiment, recall and precision values at various parameters for each dataset were used to examine the quality of LOF values produced by the grid-LOF.

**Table 1:** Experiment Datasets.

Dataset	# of instances	# of attributes
Banknote Authentication (BA)	1372	5
Wilt	4889	6
Parkinson Telemonitoring (PT)	5875	19
Combined Cycle Power Plant (CCPP)	9568	5

As discussed in the previous section, the size of the grids, determined by the interval number *k*, can influence the performance of the grid-LOF algorithm. As the interval number *k* increases the grid size decreases, which results in more calculation time and smaller approximation error. Thus, experiments were conducted with varying interval numbers, *k*.

The experiments were run on a 1.60 GHz, 4.00 GB PC. The code for implementation of both the brute force LOF and grid-LOF algorithms were written in Python.

### Improvement in search time efficiency

**Fig 3:**
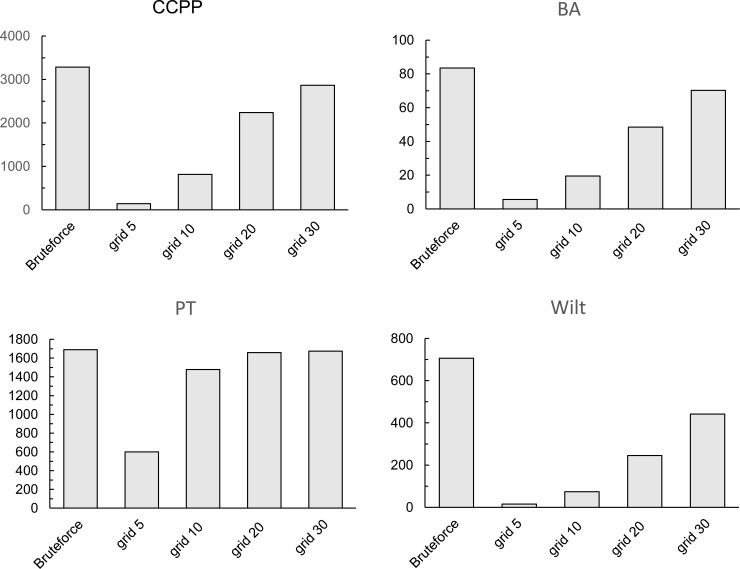
LOF calculation time (seconds) of brute-force algorithm and grid-LOF algorithm for four datasets.

Fig 3 depicts the calculation time of the original and grid-LOF algorithms for the four datasets. For each dataset, the x-axis represents different experiment scenarios with respect to interval numbers (*k* = 5, 10, 20, and 30) defined for each dimension. The y-axis represents the calculation time (seconds) of each experiment scenario. In the experiment, the number of nearest neighbors was fixed at ten. As indicated, for the four datasets, the grid-LOF algorithm was superior to the brute-force algorithm in calculation time. The time efficiency, though, diminished as the grid number increased. In particular, when the interval number was 30, the calculation time was virtually the same as the brute-force algorithm.

**Fig 4:**
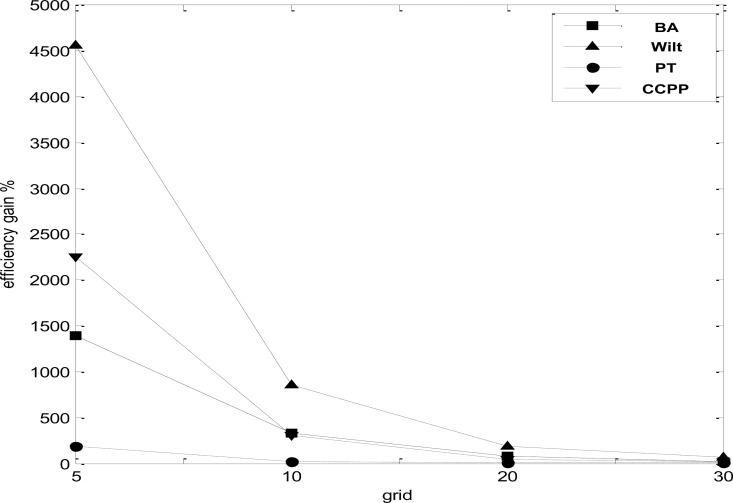
LOF calculation time of brute-force algorithm and grid-LOF algorithm for four datasets.

Fig 4 illustrates the computation time efficiency of the grid-LOF algorithm over the original algorithm. The x-axis represents the interval numbers (*k*) defined for each dimension. The y-axis represents the percent gain of the grid-LOF over the brute-force method. As indicated in Fig 4, the time efficiency gain ranged from 1388.329 (Parkinson, grid = 30) to 0.931 (Wilt, grid = 5). The efficiency gain effect was more significant with a larger grid size. The efficiency gain gradually decreased as the interval value *k* increased. A greater interval value means that the search space was subdivided into smaller grids, which resulted in less efficiency gain and less approximation errors.

Fig 4 also depicts the time efficiency gain of the four datasets. Three datasets including Banknote Authentication (BA), Wilt, and Combined Cycle Power Plant (CCPP) had a significant time efficiency gain; Parkinson Telemonitoring (PT) had a moderate gain compared to the other data sets. The moderate performance of Parkinson Telemonitoring (PT) data is related to the large attribute number (19) of the data because the grid-LOF addresses the computation complexity of the *k*-nearest neighbors, not the “Curse of Dimensionality”. Therefore, the proposed algorithm can provide improved performance for low-medium dimensional data with a large number of data points.

### Effectiveness: Quality deterioration of the Grid-LOF

**Fig 5:**
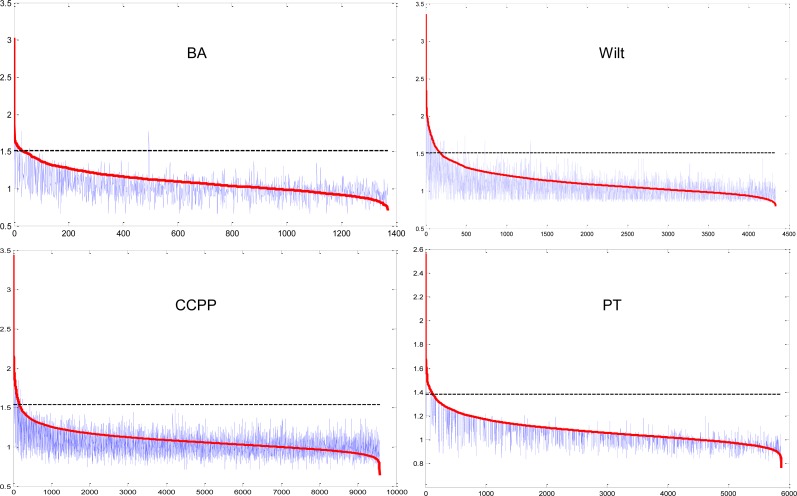
Difference between exact LOFs (brute-forced LOF) and approximated LOFs (grid-LOF).

Fig 5 depicts the difference between the exact LOFs obtained from the brute-force method and approximated LOFs obtained from the grid-LOF method for each dataset. On the horizontal axis, the data points are sorted by the exact LOFs with decreasing order. The red curve represents the exact LOFs, while the lumpy blue curve represents the approximated LOFs. As indicated in the figure, the LOFs from the grid-LOF algorithm are different from exact LOF values not only in absolute scale but also in the order of LOF values.

In this study, the quality deterioration of the grid-LOF algorithm is compared with the original algorithm in terms of precision and recall. A data point with greater original LOF value than a predetermined threshold (marked by the horizontal dotted line in Fig 5) is considered as a true outlier. Then, a data point with a grid-LOF value over the 90th percentile is selected as a potential outlier.

**Fig 6:**
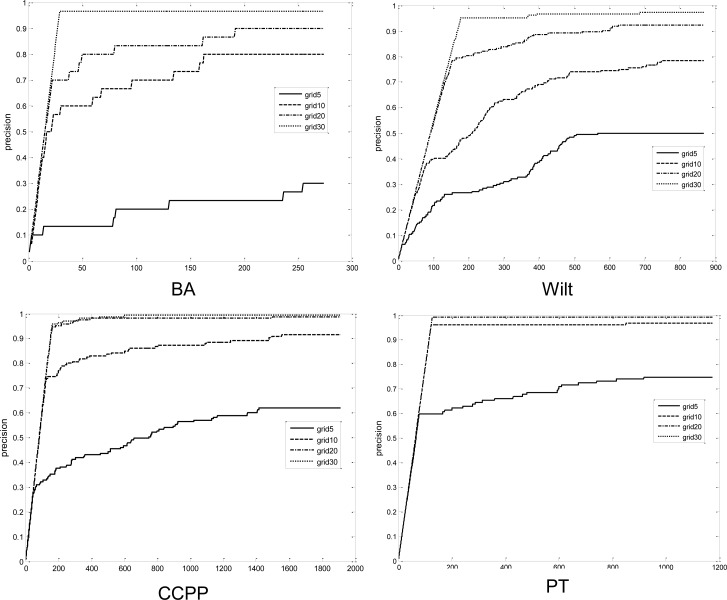
Precision of LOFs obtained from grid-LOF algorithm.

**Fig 7:**
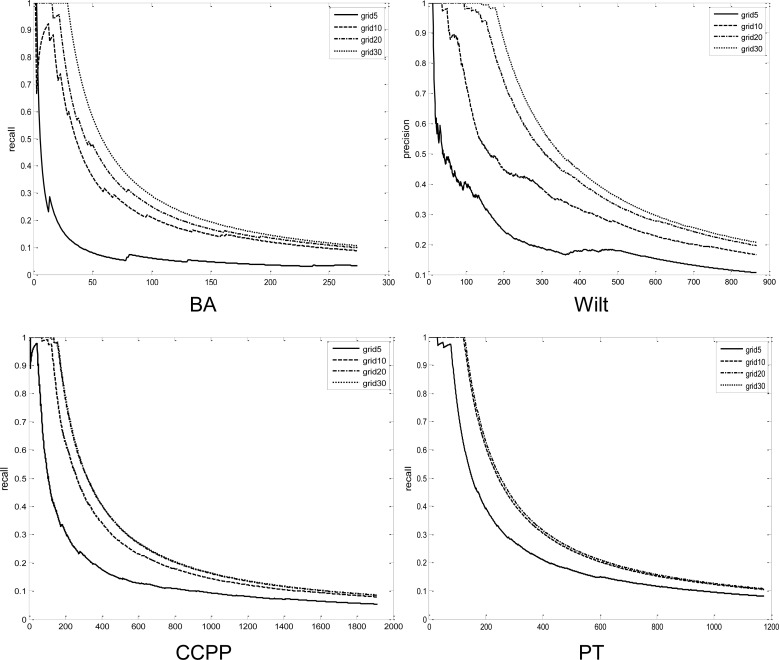
Recall of LOFs obtained from grid-LOF algorithm.

Fig 6 and Fig 7 illustrate the precision and recall with respect to different interval numbers for each dataset. As indicated, the solution quality deteriorates as a smaller number of grids are used. When the interval number is five, the recall does not exceed 0.5 until the top 10 percent grid-LOF values. This implies that virtually half of the true outliers cannot be predicted within the top 10 percent grid-LOF values. The solution quality, however, increases rapidly when the interval number increases up to ten. In all cases, more than 70 percent of the true outliers were identified within a search of the top 10 percent data. If the interval number increases to 30, there is virtually no quality deterioration in the grid-LOF method. However, as indicated in Fig 4, there is less efficiency gain when *k* = 30. Considering the trade-off between efficiency and effectiveness, a strategy that accepts a minor quality deterioration in exchange for a faster calculation is useful in situations requiring real-time outlier detection.

Fig 6 also displays the effect of the dataset on the quality deterioration. Parkinson, which has the largest number of attributes (19), indicates reasonable performance over the other datasets. This result is because the total grid number is greater than other datasets, although each dimension is divided by the same intervals. Among the datasets with similar attribute sizes (Banknote, Wilt, CCPP), CCPP outperformed the others.

## Case Study

In this section, to further validate the proposed algorithm, experiments were conducted with a real dataset with a large volume. In the present study, the database of transaction logs of KAERI (Korea Atomic Energy Research Institute) were used. The dataset consisted of 297,019 records and 33 fields. For a further description of this dataset, please refer to [14].

The raw dataset set was preprocessed to allow it to be analyzed by the grid-LOF algorithm. First, trivial data fields were manually removed. Among the 33 fields, seven fields were considered for the grid-LOF calculation. Table 2 represents the name and property of the selected fields. Then, categorical or text values were converted into numerical values because LOF calculations are based on real numbers. Moreover, each field was standardized to have a zero means and unit variance. Finally, duplicated records were eliminated from the dataset. Consequently, the preprocessed dataset contained 17,140 records with seven fields. The experiment was executed in the same environment as in the previous section. Similar to the previous experiments in Sections 3, the number of nearest neighbor was fixed at ten.

**Table 2:** KAERI dataset.

Field	Description	Preprocess
dbdatasize	Query size	No treatment
dbipfrom	Client IP	Mapping IP to integer number
dbquerytype	Type of query	Mapping from “DML”, “DDL”, “DCL” to 1, 2, 3
dbdurationtime	Response time (ms)	No treatment
dbtable	Table name used in query	Mapping from unique table name to unique integers (0,…,514)
dbcolumn	Column name used in query	Mapping from unique table name to unique integers (0,…,759)
dbcommand	Query command	Mapping from “NULL”, “ALTER”, “COMMIT”, “ROLLBACK”, “UPDATE”, “DELETE”, “INSERT”, “SELECT” to 0,…,7

First, we compared the computation time of the original and grid-LOF algorithms. Table 3 represents the calculation time of the original and grid-LOF algorithms with different grid sizes. As indicated in the table, the calculation time was reduced dramatically by the grid-LOF algorithm. The time efficiency gain ranged from 42.64 (*k* = 10) to 11.25 (*k* = 100). It is noteworthy that there was minimal efficiency loss as the grid size decreased. One possible explanation of this result is that the dataset had a dense structure where the majority of the data records belonged to a few number of grids.

**Table 3:** LOF calculation time (seconds) comparison.

Brute force	Grid 10	Grid 20	Grid 30	Grid 40	Grid 50	Grid 60	Grid 70	Grid 80	Grid 90	Grid 100
3957.3	92.8	182.6	230.5	258.5	271.8	299.5	316.0	330.1	337.9	351.5

**Fig 8:**
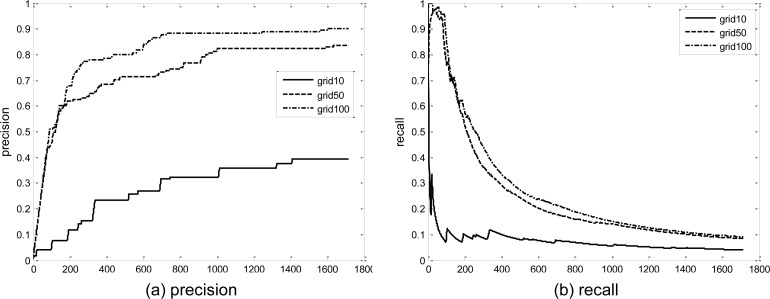
Precision and Recall of LOFs obtained from grid-LOF algorithm.

We also measured the solution quality of the grid-LOF algorithm. Data points whose original LOF values were greater than the 99^th^ percentile were determined as true outliers. Then data points whose grid-LOF values were greater than the 90^th^ percentile were considered as potential outliers. Fig 8 displays the quality deterioration with the grid-LOF methods (*k* = 5, 50, and 100) compared with the original method. As indicated in the figure, the solution quality increased rapidly as the size of the grid decreased. When *k* = 100, the grid-LOF identified almost 80 percent of the true outliers within a search of the top two percent of data. The precision rate increased up to 90 percent until the 90^th^ percentile of the dataset. The result confirms that the grid-LOF algorithm can enhance the time efficiency of outlier detection while maintaining solution quality.

## Conclusion

To reduce the LOF algorithm’s computation time required for the *k*-nearest neighbors, a grid-LOF algorithm that divides the data spaces into grids was proposed. A set of experiments incorporating various parameters was conducted. The proposed method demonstrated a significant computation time reduction with predictable and acceptable trade-off errors. Then, the proposed methodology was successfully applied to real database transaction logs.

The main advantage of the proposed algorithm lies in its computational efficiency as it calculates the LOF values based on the centroid of a grid instead of the entire set of data. Thus, for a very large dataset, the grid-LOF can be considered as an acceptable approximation for the original LOF. The efficiency gain of the grid-LOF was more significant when it was applied to the real data.

Moreover, it can also be effectively used for real-time outlier detection. If the brute-force, original LOF approach is assumed, the LOF value of every point must be recalculated and updated every time new data points are added to the data set, often resulting in poor performance. The grid-LOF can rapidly detect outlierness of new data points, as it can utilize a grid structure of existing data points. In the grid-LOF, the LOF calculation of new data points only requires the identification of the grid-location of the data points and no further calculations are required.

However, this article also addressed some limitations of the grid-LOF. As discussed in Section 4, the grid-LOF had only a moderate efficiency gain with high dimensional data. Thus, the grid LOF is more appropriate for low-medium dimensional data with a large number of data points. To further enhance the performance of the grid-LOF, the application of a dimensionality reduction method such as Support Value Decomposition (SVD) to the grid-LOF could be a suitable future research topic.

## References

[1] Aggarwal CC, Yu PS. Outlier detection for high dimensional data. In: Sellis T, Mehrotra S editors. Proceedings of the 2001 ACM SIGMOD International Conference on Management of Data; 2001 May 21–24; Santa Barbara, CA, USA. New York: ACM press; 2001. p.37-46

[2] Hawkins DM. Identification of outliers London: Chapman and Hall; 1980

[3] Pokrajac D, Lazarevic A, Latecki LJ. Incremental local outlier detection for data streams. In: Duch W, Ghosh J, editors. Proceedings of IEEE Symposium on Computational Intelligence and Data Mining (CIDM), Honolulu, Hawaii, IEEE Press, New York, p. 504–515.

[4] Breunig MM, Kriegel HP, Ng RT, Sander J. LOF: identifying density-based local outliers. In: Dunham M, Naughton JF, Chen W, Koudas N, editors. Proceedings of the 2000 ACM SIGMOD International Conference on Management of Data; 2000 May 15–18; Dallas, TX, USA. NEW YORK: ACM Press; 2000. p. 93–104.

[5] Maloof M A. Machine Learning and Data Mining for Computer Security: Methods and Applications. London: Springer-Verlag; 2006.

[6] Lazarevic A, Ertöz L, Kumar V, Ozgur A, Srivastava J. (2003). A Comparative Study of Anomaly Detection Schemes in Network Intrusion Detection. In: Barbara D, Kamath C, editors. Proceedings of the 2003 SIAM Conference on Data Mining; 2003 May 1–3; San Francisco, CA, USA. PA; Society for Industrial and Applied Mathematics. p. 25–36.

[7] Kang B, Kim D, Kang SH. Real-time business process monitoring method for prediction of abnormal termination using KNNI-based LOF prediction. Expert Syst Appl. 2012;5: 6061–6068.

[8] Kim S, Cho NW, Kang B, Kang SH. Fast outlier detection for very large log data. Expert Syst Appl. 2011;8: 9587–9596.

[9] Press WH, Teukolsky SA, Vetterling WT, Flannery BP. Numerical recipes in C London: Cambridge University Press; 1988

[10] Strang G. Linear algebra and its applications 2nd ed. Cambridge: Academic Press; 1980

[11] Fukunaga K. Introduction to statistical pattern recognition 2nd ed. Cambridge: Academic Press; 1990

[12] Faloutsos C, Lin KI. FastMap: A fast algorithm for indexing, data-mining and visualization of traditional and multimedia datasets. In: Carey M, Schneider D, editors. Proceedings of the 1995 ACM SIGMOD International Conference on Management of Data; 1995 May 22–25; San Jose, CA, USA. New York: ACM Press; 1995. p. 163–174.

[13] Blake C, Keogh E, Merz CJ. UCI repository of machine learning databases [cited 2016 June 1]. Available: http://www.ics.uci.edu/~mlearn/MLRepository.htm.

[14] Kim S, Cho NW, Lee YJ, Kang SH, Kim T, Hwang H, Mun D. Application of density-based outlier detection to database activity monitoring. Inf Syst Front. 2013;1: 55–65.

